# DMAP: a connectivity map database to enable identification of novel drug repositioning candidates

**DOI:** 10.1186/1471-2105-16-S13-S4

**Published:** 2015-09-25

**Authors:** Hui Huang, Thanh Nguyen, Sara Ibrahim, Sandeep Shantharam, Zongliang Yue, Jake Y Chen

**Affiliations:** 1 School of Informatics and Computing, Indiana University, Indianapolis, IN 46202, USA; 2 Department of Computer and Information Science, Purdue University, Indianapolis, IN 46202, USA; 3 School of Medicine, Indiana University, Indianapolis, IN 46202, USA; 4 Institute of Biopharmaceutical Informatics and Technology, Wenzhou Medical University, Zhejiang, China

## Abstract

**Background:**

Drug repositioning is a cost-efficient and time-saving process to drug development compared to traditional techniques. A systematic method to drug repositioning is to identify candidate drug's gene expression profiles on target disease models and determine how similar these profiles are to approved drugs. Databases such as the CMAP have been developed recently to help with systematic drug repositioning.

**Methods:**

To overcome the limitation of connectivity maps on data coverage, we constructed a comprehensive in silico drug-protein connectivity map called DMAP, which contains directed drug-to-protein effects and effect scores. The drug-to-protein effect scores are compiled from all database entries between the drug and protein have been previously observed and provide a confidence measure on the quality of such drug-to-protein effects.

**Results:**

In DMAP, we have compiled the direct effects between 24,121 PubChem Compound ID (CID), which were mapped from 289,571 chemical entities recognized from public literature, and 5,196 reviewed Uniprot proteins. DMAP compiles a total of 438,004 chemical-to-protein effect relationships. Compared to CMAP, DMAP shows an increase of 221 folds in the number of chemicals and 1.92 fold in the number of ATC codes. Furthermore, by overlapping DMAP chemicals with the approved drugs with known indications from the TTD database and literature, we obtained 982 drugs and 622 diseases; meanwhile, we only obtained 394 drugs with known indication from CMAP. To validate the feasibility of applying new DMAP for systematic drug repositioning, we compared the performance of DMAP and the well-known CMAP database on two popular computational techniques: drug-drug-similarity-based method with leave-one-out validation and Kolmogorov-Smirnov scoring based method. In drug-drug-similarity-based method, the drug repositioning prediction using DMAP achieved an Area-Under-Curve (AUC) score of 0.82, compared with that using CMAP, AUC = 0.64. For Kolmogorov-Smirnov scoring based method, with DMAP, we were able to retrieve several drug indications which could not be retrieved using CMAP. DMAP data can be queried using the existing C2MAP server or downloaded freely at: http://bio.informatics.iupui.edu/cmaps

**Conclusions:**

Reliable measurements of how drug affect disease-related proteins are critical to ongoing drug development in the genome medicine era. We demonstrated that DMAP can help drug development professionals assess drug-to-protein relationship data and improve chances of success for systematic drug repositioning efforts.

## Background

To reposition drugs [1-3] from one approved indication to a new indication, drug developers could significantly save associated development cost [4] and lower development risks[5]. With the rapid accumulation of genomics, functional genomics, and chemical informatics data in the past decade, several new systematic approaches to drug repositioning have been proposed. For example, one may study the drug-ligand structural binding relationships systematically for all approved drugs to discover their new targets implicated in other diseases using chemoinformatic tools [6]. If the drug-drug similarity relationships, disease-disease similarity relationships, or side-effect-to-side-effect similarity relationships [7] are characterized, one may populate indications from one drug to another among all drugs under study that are closely related through shared disease, shared side effect, or shared target relationship profiles. Machine learning [1] and biomedical literature text mining [8] approaches can also help uncover non-obvious relationships between approved drugs and potential new indications.

Recently, there has been surging interest to apply "connectivity map" (CMAP) techniques, which attempt to match a repositioned drug's effects by their shared disease perturbation gene expression profiles [2,3,9-11]. A major resource--CMAP--was developed by Lamb et al. [11] to assay genome-wide transcriptional expression data across a wide range of cell lines treated with small drug molecules. Based on the CMAP data, Iorio et al. [3] proposed a drug repositioning method by constructing drug-drug similarity networks. Hu and Agarwal[9] and Sirota et al. [2] also investigated how to pair drugs and disease indications based on negative correlation of drug perturbation and disease gene expression patterns identified from CMAP. The anti-correlation relationships between the drugs and diseases are demonstrated to suggest novel therapeutic indications for existing drugs. The primary advantage of CMAP is that it does not require prior knowledge of drug targets or a drug's detailed mechanism of actions to work. However, CMAP's limitation is also quite apparent: limited coverage of drugs, limited drug perturbation gene expression data, limited dosage-dependent conditions, and the dubious transferability of expression patterns from cell lines or animal models to human systems. Ultimately, it can be time-consuming and costly before a significant portion of current drugs in all safe dosage conditions can be tested in even a limited number of cell lines for CMAP according to the statistics in [12].

**Figure 1:**
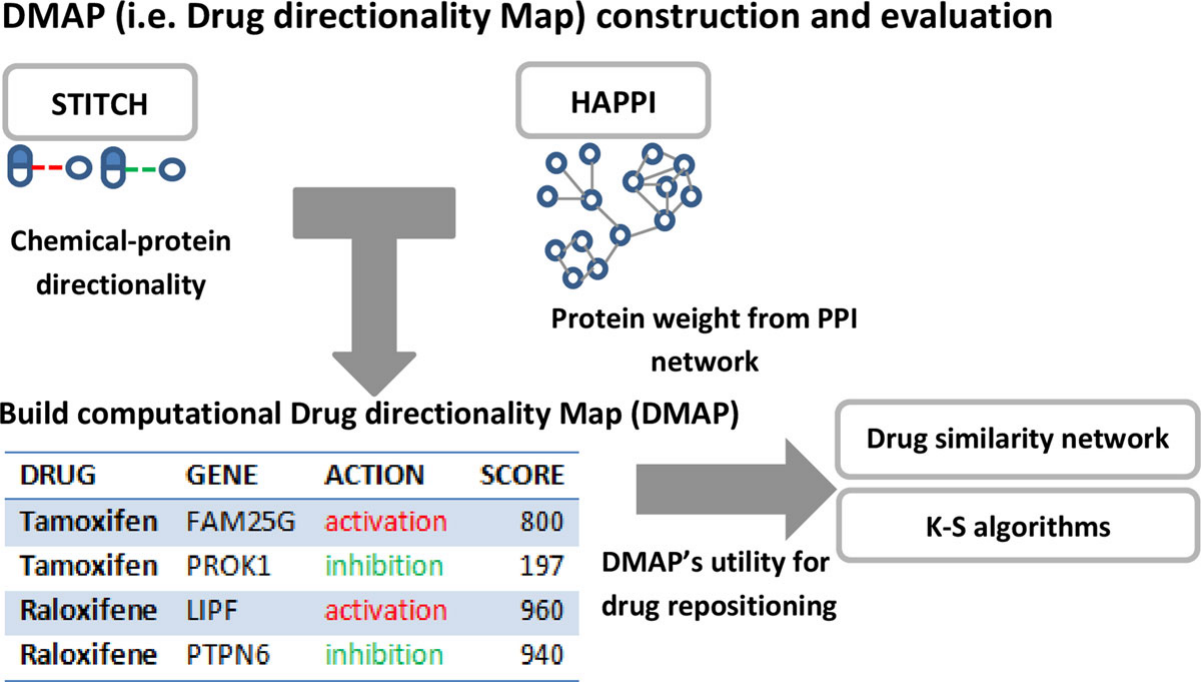
**Computational framework**. Construct the Drug directionality Map (DMAP) dataset from STITCH and HAPPI and evaluate DMAP's utility for drug repositioning with drug similarity network and K-S algorithms.

In this work, we describe our development of a new resource called DMAP that can help drug development researchers evaluate what effects a drug may have on disease-relevant genes or proteins. DMAP compiles each drug's stimulatory or inhibitory effects on genes or their protein products (Figure 1), based on the computational integration of such data from different databases. It covers 438,004 chemical-to-protein effect relationships between 24,121 PubChem compounds that cover 289,571 chemical entities with a synonymous name, and 5,196 distinct UniProt proteins. DMAP may be used wherever CMAP data coverage is poor for drug repositioning applications. To evaluate the DMAP performances, we calculated drug-to-drug similarity based on newly generated DMAP profiles [3] and obtained Kolmogorov-Smirnov test scores [2,11]. We demonstrate that DMAP can successfully recall known drugs for examined disease indications. In addition, by applying DMAP, we propose novel indications for drugs currently in NCATS [13].

## Methods

### Develop DMAP context from existing databases

In DMAP, we collected, integrated and ranked each pair of drug-to-protein/gene relationship. The primary data for drug-protein information comes from the STITCH [14] database, and may be expanded easily to include other sources such as CTD [15] data. STITCH is an aggregated Cheminformatics database of chemical-to-protein interactions connecting over 300,000 chemicals and 2.6 million proteins for many species mined from biomedical literature. We parsed out STICH chemical protein interactions for Homo sapiens with those chemical-protein "edge actions" being either "activation" (stimulatory interaction) or "inhibition" (inhibitory interactions). To eliminate the synonymous chemicals with the same chemical structure, we mapped 289,571 chemicals to the PubChem database in the result of 24,121 distinct PubChem Compound ID (CID).

Next, we calculated a probability-weighted summary of all the evidence to determine an overall mechanism of "edge action" for each specific chemical-protein interaction using *conf(d,p)*.

(1) $$\text{conf}\left(\text{d},\text{p}\right)=\sum_{i=1}^{i=N}\left(prob_{i}\left(d,p\right)*sign_{i}\right)$$

where *d *and *p *are specific drugs and proteins, respectively. *N *is the number of evidence for the interaction between *d *and *p. prob_i_*(*d,p*) is the confidence of each evidence *i *with a value within the range of 0[1]. *sign_i _*has a value of 1 if the evidence *i *represents activation while has a value of -1 if the evidence *i *represents inhibition.

Then, to rank each interaction, we used HAPPI [16], an integrated protein interaction database that comprehensively integrated weighted human protein-protein interaction data from HPRD, BIND, MINT, STRING, and OPHID by assigning a weight *weight(p) *for each drug's interacting proteins using the following formula adapted from [17].

(2) $$weight\left(p\right)=k\times ln\left(\sum_{q\in NET}conf\left(p,q\right)\right)-ln\left(\sum_{q\in NET}N\left(p,q\right)\right)$$

Here, *p *and *q *are proteins on the protein interaction network, *k *is an empirical constant (*k*=2 in this study), *conf(p, q) *is the confidence score assigned by HAPPI to each interaction between protein *p *and *q*, and *N(p, q) *holds the value of 1 if protein p interacts with q or the value of 0 if protein p does not interact with q.

Finally, we developed an intuitive pharmacology score (**P-Score**) to combine the probability for each interaction and the weight of the interacting proteins:

(3) P - Score(d,p)=conf(d,p)×weight(p)

Here, P-Score contains both the information of each drug's action on their interacting proteins and the importance of the protein in the protein-protein interaction network. This is different than the expression level based ranking in CMAP, which may be more suitable for biomarker discovery instead of drug discovery. With P-Score for each drug-protein interaction, DMAP is thus in a compatible format with CMAP [11].

### Integrate drug therapeutic indication data

To construct a golden standard of known drug indications to evaluate DMAP's drug repositioning performance, we integrated the Therapeutic Target Database (TTD) [18] and the dataset from the PREDICT [1] paper. TTD is a database that provides information about drugs' known therapeutic protein targets and their targeted diseases. The PREDICT paper provides a compiled list of drug indications. We integrated these two sources to get 2,912 drug indication associations corresponding to 1,180 drugs and 726 indications.

### Prepare disease expression signatures and drug expression signatures

To apply the Kolmogorov-Smirnov algorithms with DMAP or CMAP for the drug repositioning, we need the disease expression dataset as one of the inputs. We thus retrieved the disease gene expression profiles from Pacini C et al. [19]'s paper. In total, 87 disease associated microarray experiments were compiled to represent 45 distinct diseases. According to Pacini C's paper, these datasets were obtained from the GEO microarray repository [20]. The raw CEL files were normalized with RMA [21]. For those gene expression profiles representing the same disease, they were combined with the median rank normalization by Warnat et al. [22].

The drug-gene expression datasets were obtained from Iorio et al.[3]'s paper instead of directly from CMAP [11] to reduce the batch effect. Iorio et al.[3] computed a single synthetic ranked list of genes, called Prototype Ranked List (PRL), by merging all the ranked list of the same compound in CMAP. Only consistently overexpressed/underexpressed genes are placed at the top/bottom of the RPL. This helped capture a consensus transcriptional response for each drug. We thus chose to use the PRL to represent the drug signatures from CMAP in this study.

### Design drug similarity measurement

The hypothesis for the drug similarity network approach is: if two drugs were similar, the disease indication for one drug could be potentially assigned to the other drug. To measure the similarity among each drug pair, we computed *SIM(d_x,_d_y_) *based on the Tanimoto Coefficient between their interacting proteins (4).

(4) $$SIM\left(d_{x},d_{y}\right)=\frac{\left|{\text{p}}_{\text{x}+}\cap {\text{p}}_{\text{y}+}|-|{\text{p}}_{\text{x}-}\cap {\text{p}}_{\text{y}-}\right|}{\left|{\text{p}}_{\text{x}}\cup {\text{p}}_{\text{y}}\right|}$$

Here, *d_x _*and *d_y _*represent the two specific drugs, *p_x _*represents the set of proteins interacting with *d_x_, p_y _*represents the set protein interacting with *d_y_*. |*p_x _*∪ *p_y_*| is the number of total distinct proteins in *p_x _*and *p_y_*. |*p_x+ _*∩ *p_y+_*| is the number of overlapped proteins on which both drugs have identical interactions (i.e. both activate or inhibit the shared proteins). |*p_x- _*∩ *p_y-_*| is the number of shared related proteins on which the two drugs have opposite interactions (i.e. one activates while the other inhibits the shared proteins). *SIM(d_x,_d_y_) *lies in the range of [-1,1] with 1 representing that the two drugs share the same interacting proteins and the drugs' action on each protein is the same while -1 representing that the two drugs share the same proteins but the drugs' action on each protein is opposite.

### Evaluate the prediction performance

**Figure 2:**
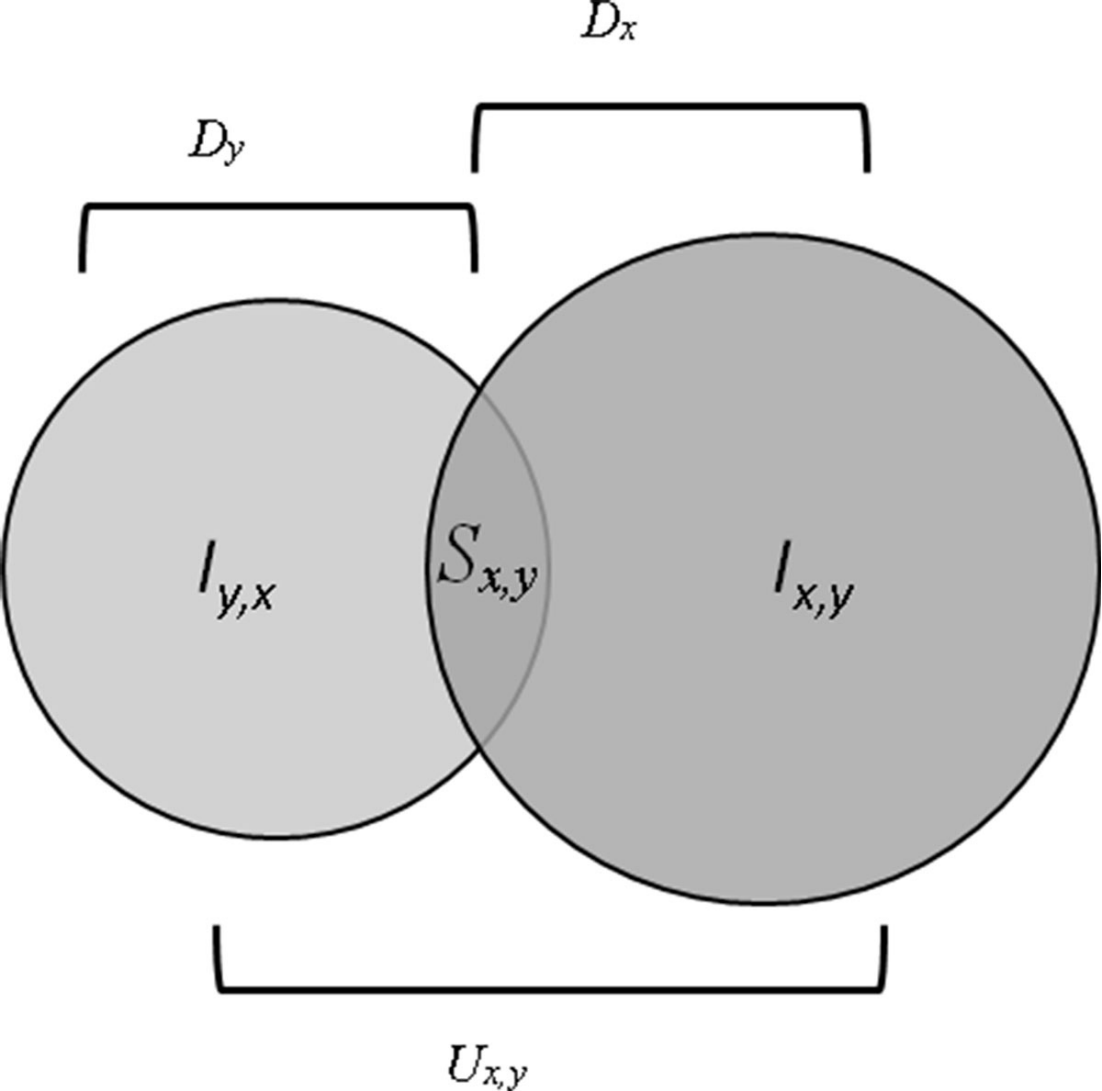
**A schematic representation of the GBA method**. Given two drugs × and y and their corresponding indication profiles Ix and Iy, respectively, the potential novel uses for drug × is Iy,x. Similarly, potential novel drug uses for drug y is Ix,y.

To assess the prediction performance, we implemented the 'Guilt by Association' (GBA) concepts (Figure 2.) presented by Chiang et al[23] and conducted "Leave-One-Out" cross-validation. For each drug, we removed its known indications and attempted to recover them by considering the indications for its top N similar drugs found. We calculated overall sensitivity and specificity by varying N--the number of similar drugs--from 1 to 981. The area under the ROC curve (AUC) score was used to measure the performance.

### Implement Kolmogorov-Smirnov strategy

We implemented the nonparametric, rank-based strategy based on the algorithm originally introduced by Lamb et al.[11] to generate a ranked list of candidate drugs for each disease. For each disease signature, we computed an enrichment score separately for the up- or down- regulated genes: es_up _and es_down_. In specific, we constructed a vector V of the position of each of the up- or down- regulated genes on the basis of the values from the reference drug dataset. The vector was then sorted in ascending order such that V(*j*) is the position of disease gene *j*. The computation of the enrichment score is based on Kolmogorov-Smirnov statistic and the details can be referred to in the supplementary material in Lamb et al. [11]. The drug score is set to zero, where es_up _and es_down _have the same algebraic sign. Otherwise, we set the drug score to es_up_-es_down_. To evaluate the statistical significance of the score, we applied a permutation approach by randomly selecting any drug signatures and re-calculated the score accordingly. We did the permutation 200 times for each drug-disease pair and computed the *p-value *by checking the actual score with the score distribution after randomization. We hypothesized that those drugs with a statistically significant negative score might be a possible treatment for the disease of interest.

### Perform literature validation

To check whether the predicted drug-disease pairs have clinical literature evidence, we used the esearch API provided by NCBI. The query term we used is '*drug name *AND *disease name *AND (Clinical Trial[ptyp] OR Clinical Trial, Phase I[ptyp] OR Clinical Trial, Phase II[ptyp] OR Clinical Trial, Phase III[ptyp] OR Clinical Trial, Phase IV[ptyp])'. We recorded the total number of clinical type PubMed articles for each association.

## Results and discussion

### Drug directionality Map (DMAP) Construction

**Figure 3:**
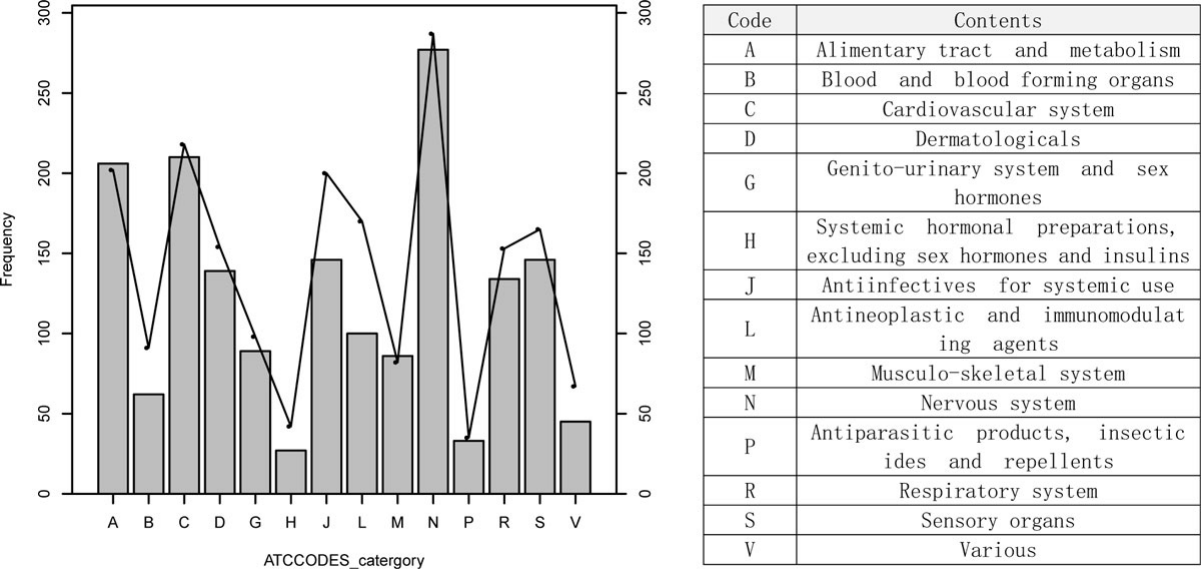
**ATCCODES distribution of DMAP database, the background is the Drugbank approved drug distribution**.

**Figure 4:**
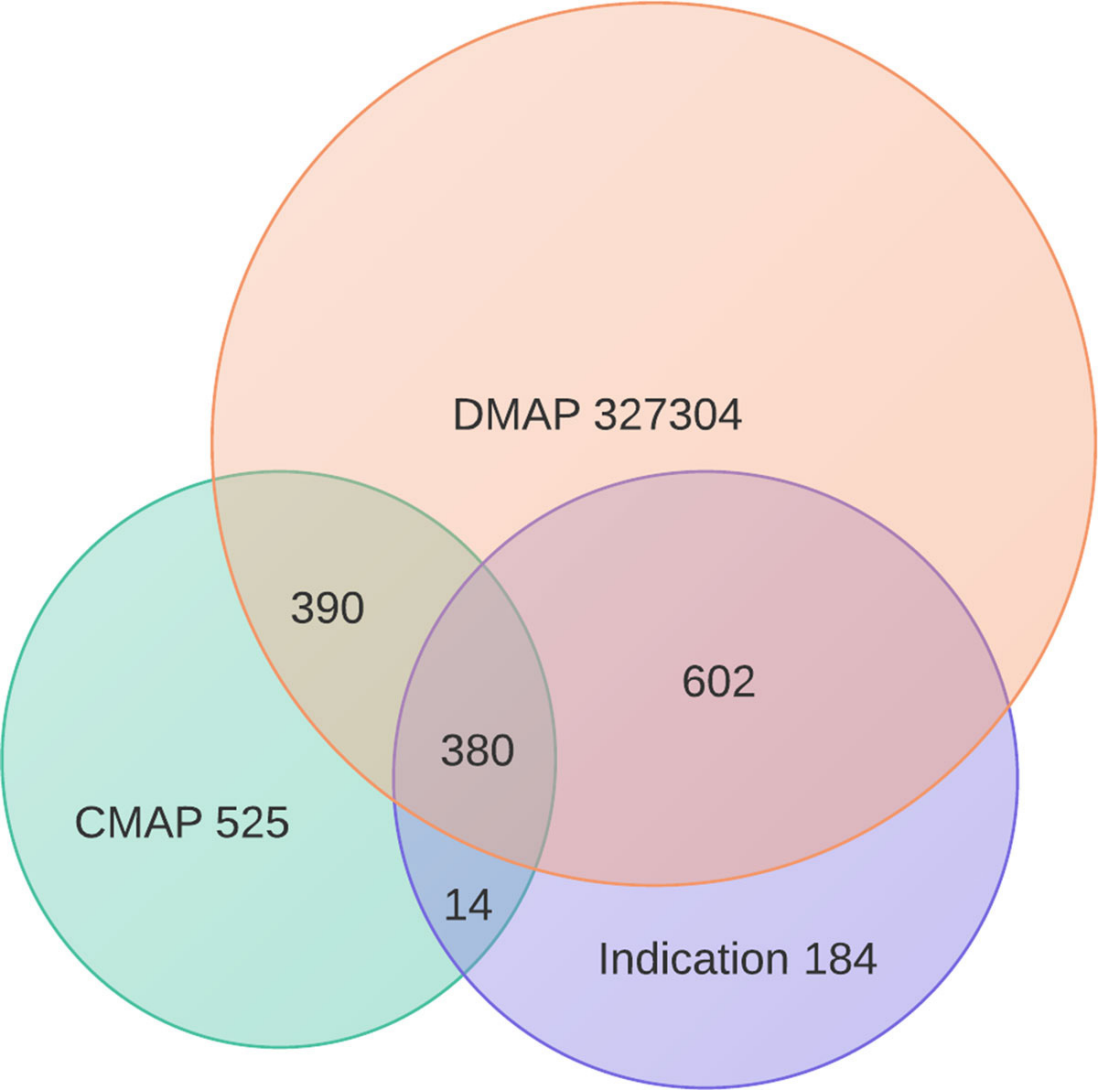
**The Venn diagram of drugs from DMAP drug signatures, CMAP drug signatures and drugs with Indication (acquired from TTD database)**.

We constructed DMAP containing 438,004 chemical protein interactions for 24,121 PubChem Compound. (Table 1). Compare to CMAP [11], DMAP shows a 14-fold increase of CID coverage. In addition, DMAP cover most of the Anatomical Therapeutic Chemical (ATC) categories: 100% at the first level, 94.3% at the second level and 92.% at the third level. This fact is significant if we compare CMAP coverage on ATC categories: 100% at the first level, 12.5% at the second level and 11.7% at the third level. Comparing to all the drugs from DrugBank, we have 71.5% in approved group and 12.7% in experimental group in DMAP that exceeding the 39.2% and 11.7% respectively in CMAP. The protein and drug-protein interaction coverage in DMAP does not match to CMAP due to CMAP is based on the whole protein screening. The DMAP most popular ATC category distribution is general balance with the DrugBank approved drug distribution except for infectious disease and cancer drugs in category J and L in Figure 3. The reason for the approved drugs in Drugbank little higher rate than DMAP in J and L is probably due to the favor of infectious disease and cancer drug discovery in Drugbank. We also normalize the score range [-1,1] between CMAP and DMAP to perform Pearson correlation and the result is 0.017, which indicates the difference exists between the cell line drug-protein result, and the protein-chemical association and predicted drug-downstream target association in human. Figure 4 shows the number of shared chemicals between DMAP, CMAP, and for drugs with known indications which we compiled from the TTD database[24] and literature [1] (Figure 4). CMAP contains 394 drugs with known indications. Meanwhile, DMAP contains 982 drugs with known indication. Among these, CMAP and DMAP share 380 drugs, which cover 96.5% of CMAP but only 38.70% of DMAP. CMAP only contains 14 drugs not covered in DMAP; meanwhile DMAP contains 602 drugs not covered in CMAP. Thus, we argue that DMAP provides a valuable resource for repositioning existing drug for new uses. To demonstrate this, in the following section we applied two representative drug-repositioning methods with DMAP dataset and proved its utility for computational drug repositioning.

**Table 1:** Database statistics comparing CMAP (build 02) and the new DMAP.

Count	CMAP (Build 02)	DMAP
**Chemical entities (including brand names)**	1,309	289,571

Drugs with known indications	394	982

**Drug entities with unique PubChem CID**	1,714	24,121

Drugbank Approved (and %)	569 (32.3%)	1260 (71.5%)

Drugbank Experimental (and %)	51 (1.0%)	646 (12.7%)

**Coverage of Drug's Therapeutic Areas**	881	1,700

ATC first level categories (and %)	14 (100%)	14 (100%)

ATC second level categories (and %)	72 (81.8%)	83 (94.3%)

ATC third level categories (and %)	152 (76.7%)	184 (92.9%)

**Proteins by UniProtID (and %)**	11,820 (58.5%)	5,196 (25.7%)

**Drug-to-protein effect relationships**	20,242,271	438,004

Stimulatory effects	10,156,011	200,310

Inhibitory effects	10,086,260	237,694

**Figure 5:**
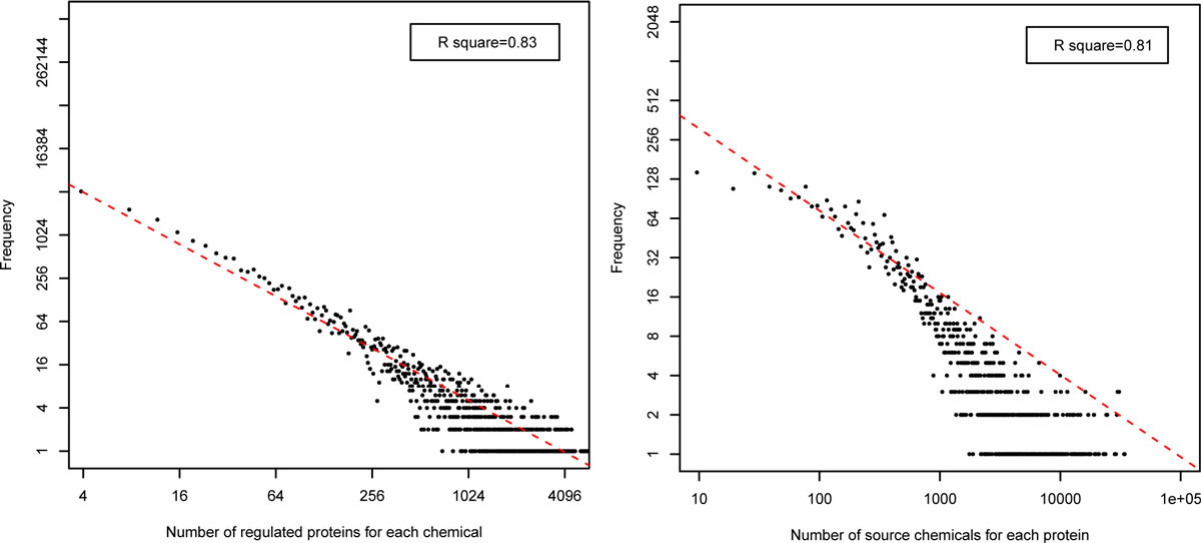
**Distribution of protein degrees (A) and drug degrees (B) in DMAP bipartite network**.

In Figure 5, we show the scale-free characteristics of the drug-protein interaction bipartite network in DMAP. Here, the drug degree of a drug is defined as the number of proteins interacting with the drug, and the protein degree of a protein is defined as the number of drugs interacting with the protein. The R-square for linear regression in drug degree and protein degree are 0.83 and 0.81 (in log scale), correspondingly.

**Figure 6:**
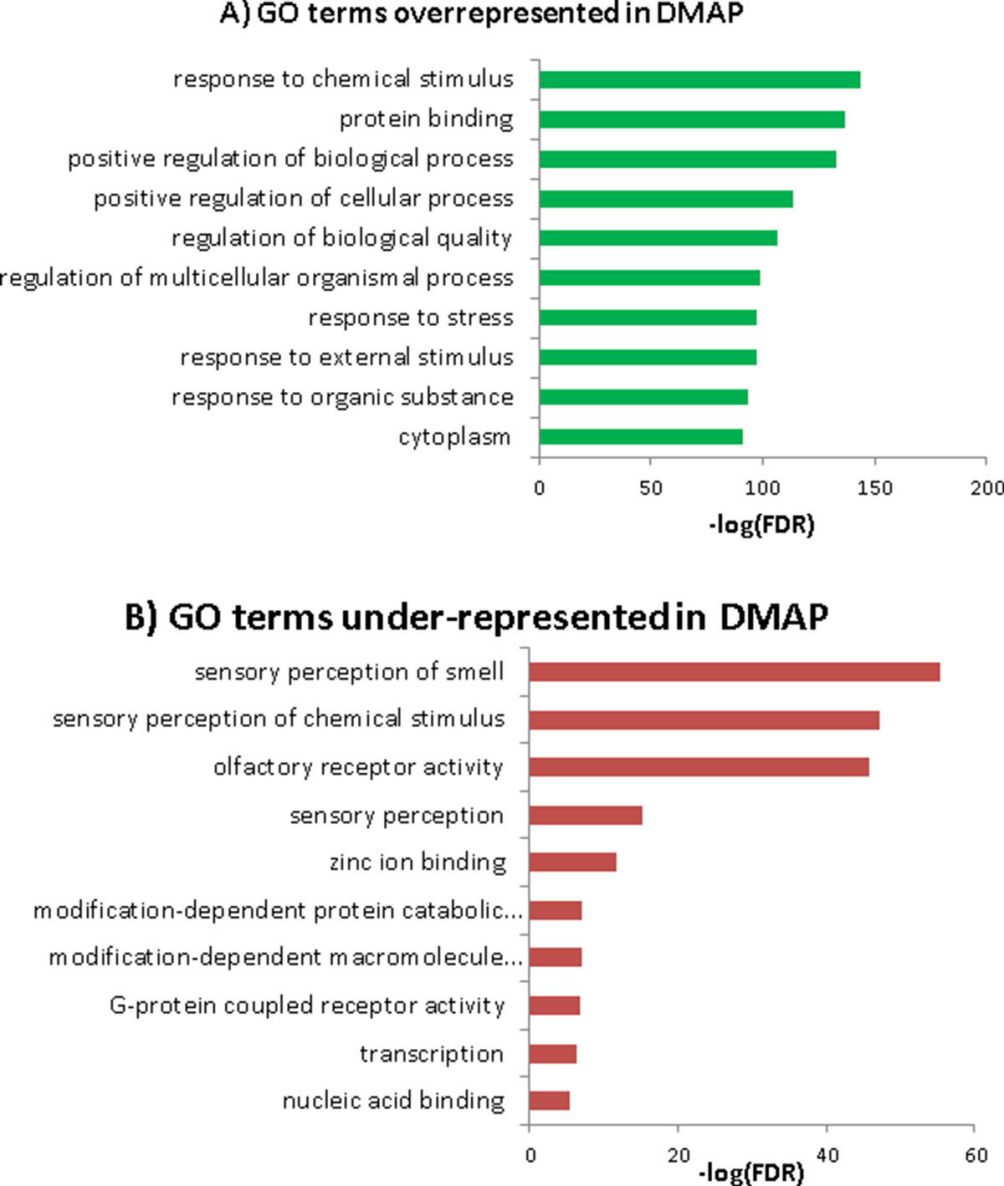
**Top 10 gene ontology terms over-represented (A) and under-represented (B) covered by DMAP's proteins**. The × axis is the -log(FDR) of gene ontology analysis acquired from DAVID functional annotation tool.

Figure 6 shows the gene ontology (GO) terminologies overrepresented (Figure 6A) and underrepresented (Figure 6B) by protein covered in DMAP. Here, we use the FDR calculated by DAVID functional annotation tool [25] on GO to sort the GO terms, and use protein not covered in DMAP to construct the underrepresented GO terms. We observe that GO 'respond' and 'regulation' terms are the most represented in DMAP.

### DMAP's utility for drug repositioning

To check DMAP's utility for drug repositioning, we applied the following two well-known drug-repositioning methods in literatures: (i) drug similarity approach [3], (ii) Kolmogorov-Smirnov algorithms [11].

### DMAP outperforms CMAP in repurposing using drug similarity approach

We computed 481,671 pairwise drug similarities for the 982 drugs with known indications by calculating the Tanimoto Coefficient between their interacting proteins profiles and evaluate the prediction performance with "Leave-One-Out" cross-validation.

We observe that using the drug-protein interaction in DMAP, the repurposing performance significantly increases, compared to the performance using the same type of information in CMAP. The Overall AUC for the prediction based on DMAP achieved 0.82. Most importantly, early retrieval performed well, with a partial AUC of 0.72 for a specificity of 90% or above[26]. Since one could only test the limited number of drugs in experimental setting, the good performance in high specificity region, approximately corresponding to the top ten candidates of all the predictions, would make the proposed drug repositioning more meaningful in practice.

**Figure 7:**
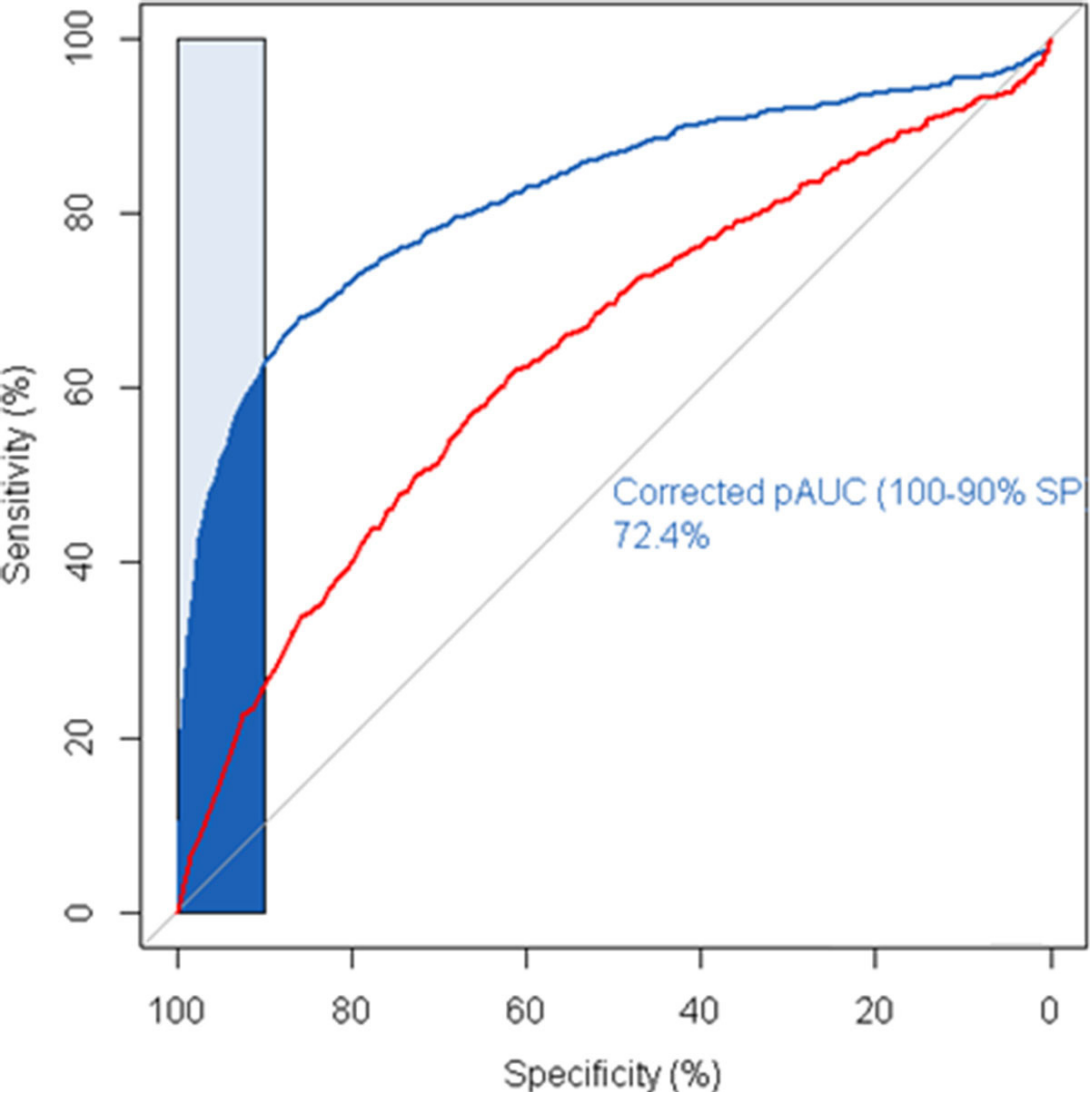
**ROC curves for the prediction performance based on DMAP (blue line) and CMAP (red line)**. Blue shade area provides a partial ROC area corresponding to specificity 90% above.

In comparison, we performed similar analysis based on CMAP transcriptome data and the overall AUC was 0.64. The early retrieval performance was only 0.55. Figure 7 showed that the ROC curve based on DMAP was above the curve from CMAP.

**Figure 8:**
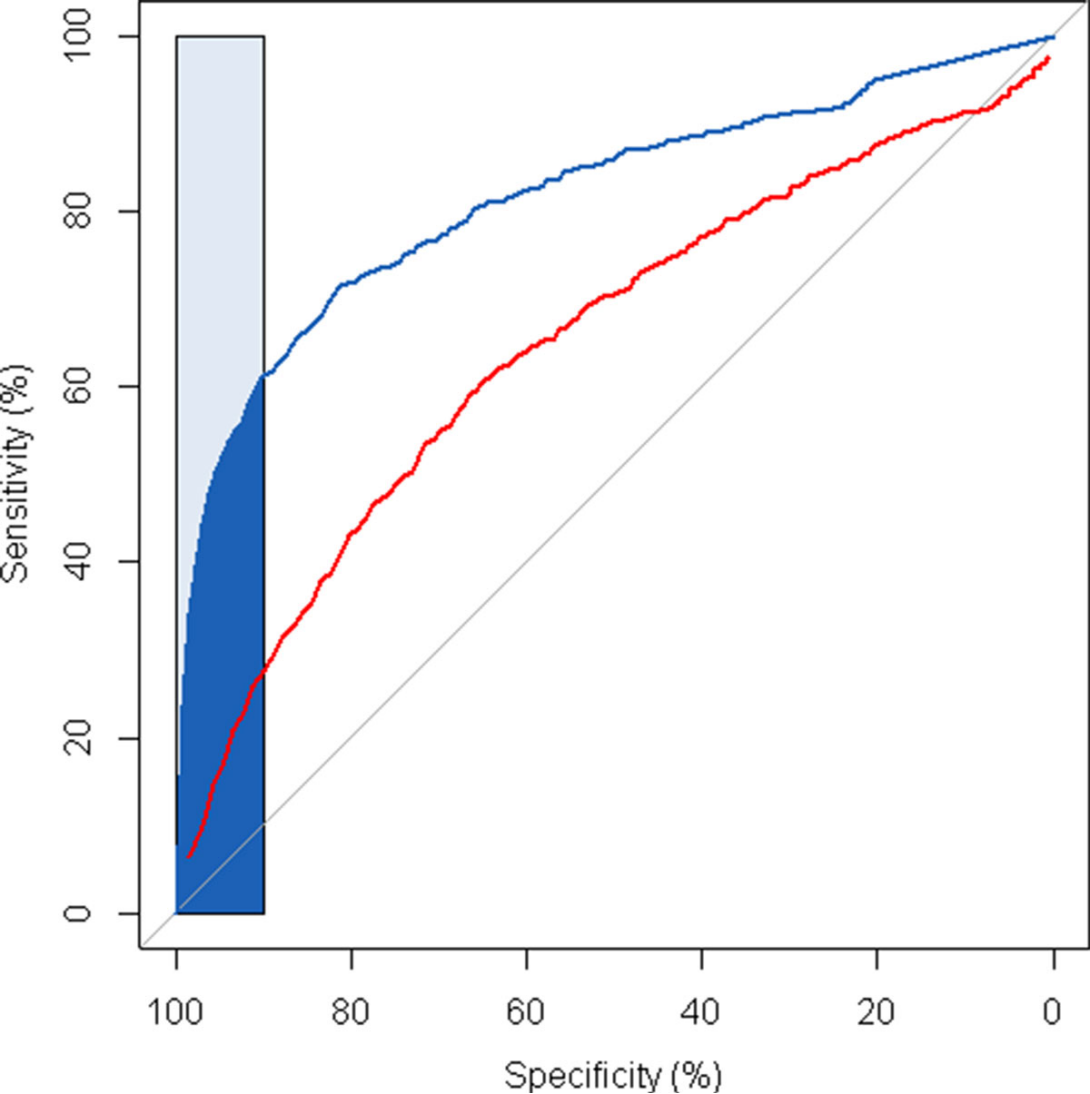
**The ROC curves for DMAP and CMAP using the overlapped drugs**.

To rule out the possibility that the performance difference was purely due to the drug coverage difference between DMAP and CMAP, we conducted the ROC analysis with only the shared drugs between DMAP and CMAP. The DMAP achieved an AUC of 0.82 while CMAP only achieved an AUC of 0.64 (Figure 8).

**Figure 9:**
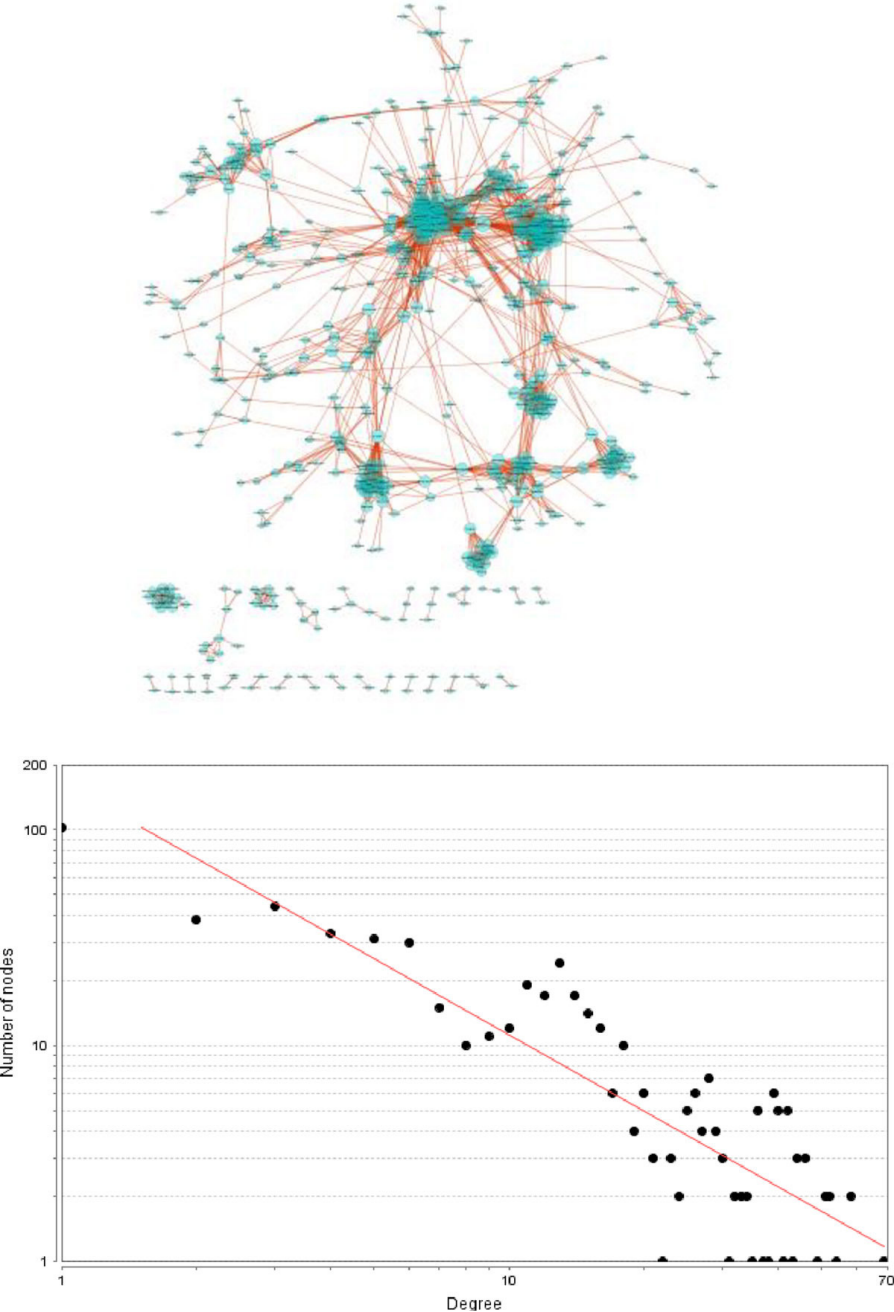
**Power-law degree distribution of drug similarity network**.

Out of all the possible drug pairs, we identified 3,014 significant pairs by requiring the number of overlapped proteins to no less than two and the drug similarity score at the top 5% of the distribution. The resulting drug network showed a scale-free property (Figure 9), commonly observed in a biological network. Most of the drugs are well connected and formed communities. In fact, 451 drug pairs out of these 3,014 significant pairs have shared at least one known disease indication. For the remaining 2,563 pairs without overlapping indications, the novel drug-disease associations from 1,206 drug pairs were supported by at least one clinical type PubMed article. Table 2 lists the top 20 drug-disease pairs and could be a good starting point for further experimental validations.

**Table 2:** Top 20 novel drug repositioning candidate identified and the count of PubMed publication support the proposed clinical indications

Drug	Disease	PubMed (Clinical)
Rocuronium	Pain	126

Clemastine	Allergies	80

Mometasone	Asthma	78

Nicotinamide	Alzheimer Disease	45

Sotalol	Hypertension	42

Sertraline	Alzheimer Disease	40

Ifosfamide	Leukemia, Acute Myeloid	40

Gabapentin	Anxiety disorder	33

Vinorelbine	Prostate Cancer	32

Lumiracoxib	Pain	28

Hydrocodone	Anesthetic	25

Zileuton	Inflammatory diseases	20

Irbesartan	Cardiovascular disease	17

Moclobemide	Parkinson Disease	13

Fluvoxamine	Alzheimer Disease	10

Ranolazine	Dysrhythmias	6

Trihexyphenidyl	Depression	5

Nicotinamide	Breast Cancer	5

Methylphenidate	Obesity	5

Pemetrexed	Colon cancers	1

#### DMAP outperforms CMAP in repurposing using Kolmogorov-Smirnov approach

We compiled the gene expression profiles for 45 distinct diseases and then queried them against DMAP and CMAP, respectively, to generate a ranked list of potential treatments for each of the diseases of interest. By using DMAP drug-protein interaction data, we were able to correctly retrieve the drugs' indications, which were unable to be retrieved using CMAP drug-protein interaction data. We examined results for diseases that are the leading causes of death in the US [27]. For breast cancer, with the DMAP, we successfully retrieved Anastrozole, Capecitabine, Doxorubicin, Estradiol, Megestrol, Paclitaxel, Testosterone and Testolactone as possible therapeutic drugs for breast cancer. With the CMAP data, only Paclitaxel was retrieved as a potential therapeutic drug. For lung cancer, we retrieved Cisplatin and Etoposide by using the DMAP. However, when CMAP was used, we were not able to retrieve any drugs for lung cancer. Additional file 1 also contains the results for other diseases. To have statistical significance, we required a *p-value *of less than 0.05. CMAP did relatively better in the case for Alzheimer's disease and Leukemia. For these known relationships covered in CMAP but not DMAP, or vice-versa, some were due to having a borderline *p-value *while others were due to violating our hypothesis of negative correlation. Overall, DMAP and CMAP database were complimentary to each other.

Besides recalling the known drug-disease relationships, with DMAP, the Kolmogorov-Smirnov approach could also propose novel drug-disease associations. National Center for Advancing Translational Sciences (NCATS) [13] provides a list of drugs for translational medical research. We cross checked the novel predictions with their drug list. Here, we highlight three case studies for Vincristine, Nifedipine and Progestrone. Vincristine is a drug typically indicated for Leukemia and Wilm's tumor. A recent study performed by Indolfi *et al.*[28] revealed that there is a potentially higher rate of survival in patients with bilateral Wilm's tumor when patients are given a dosage of vincristine/actinomycin D. Nifedipine is indicated to treat high blood pressure and angina. The DMAP results suggest that Nifedipine can also be used to treat asthma. Since Nifidipine is a PKC inhibitor and PKC is a potential therapeutic target for asthma [29], it is a potential treatment for asthma. Cheng et al [30] demonstrated in their study that Nifedipine can help control the constriction involved in sensitized tissue in asthma. Furthermore, another study by Barnes et al[31] suggested that Nifidipine modifies exercise-induced asthma. Progesterone is a prescription drug used for women taking estrogens after menopause and is also used for treating amenorrhea. The DMAP results suggest that progesterone can be used to treat breast cancer. In the study by Groshong et al [32], it was determined that treatment with Progesterone can be used to regulate Breast Cancer cell growth.

Additional file 2 summarized all the novel drug repositioning predicted by both similarity approach and KS algorithms, which could be a starting point for further experimental validation.

## Conclusions

Reliable measurements of how drugs affect disease proteins is critical to drug repositioning. In this work we presented a computational drug directionality resource called DMAP to address the challenges. We demonstrated that the resource can greatly facilitate the drug discovery process for the following reasons: access to disease gene-drug relationship data with high coverage and quality; incorporating prior knowledge about biological significance with protein interaction network.

This study differs from previous research in that it provides a comprehensive database of computationally derived drug-protein relationships. Previous efforts [2,3,9,10] on pairing the expression of drugs and diseases mainly rely on experimental connectivity map. For example, Sirota et al.[2] performed a large-scale integration of expression signatures of human diseases from the public data with CMAP drug signatures. This work provides another alternative resource of directed drug-protein relationships. The drug similarity study proves the validity of the probabilistic-based directionality for each drug-protein relationship. The implementation of K-S algorithm proves the compatibility of the pharmacology score based ranking with the expression based ranking in CMAP for the drug repositioning research. With these two major drug repositioning approaches, the knowledge base from DMAP performed better than directly using the microarray data from CMAP. It can thus serve as a valuable resource for drug repositioning studies.

One limitation of DMAP lies in that the number of interacting proteins for each drug is not a constant number. For the gene expression based profiles in the CMAP database, each drug was measured against the same number of proteins in experiments while in DMAP the number of interacting proteins varies from drug to drug. In DMAP, 13,717 drugs have at least 10 activated and inhibited proteins. Despite this limitation, the database served its purpose for systematic drug repositioning as demonstrated in this work.

Another limitation of DMAP is the dependency of drug-protein interaction scoring on protein-protein interaction (PPI) databases. As mentioned in [33], disease gene ranking should be performed using PPI data not only with reasonable quality but also high data coverage. In this work, we only used the PPI data to calculate the protein weight. Therefore, we believe the conclusions above still hold. In other words, we expect time and PPI quality to affect primarily drug-protein data significantly if and only if the drug-protein relationship score is relatively low; when the drug-protein relationship is high - suggesting that there're lots of data coverage for the relationships across many literature reports - the time or PPI quality effect is expected to be relatively small.

## Competing interests

The authors declare that they have no competing interests.

## Authors' contributions

HH performed database construction, drug repositioning performance evaluations and predictions, and led the writing of the manuscript under the guidance of JYC. TN, SI, ZLY and SS participated in data analysis and helped with documentation of the case studies. JYC conceived the project, supervised the entire research team with frequent feedback in the design, implementation, and evaluation of the project, and revision of the manuscript. All authors contributed to the completion of the manuscripts.

## Funding

Publication of this article was funded in part by the National Institute of Health to Dr Jake Chen (co-PI of R21CA173918).

## Supplementary Material

Additional File 1 **Retrieval of known disease drug relationships from DMAP and CMAP, respectively**. Click here for file

Additional File 2 **Drug repositioning predicted by both similarity approach and KS algorithms**. Note: in both files, the star rating is labelled according to the following criteria:
K-S Score<-0.3: «««««
-0.3 ≤ K-S Score<-0.2: ««««
-0.2 ≤ K-S Score<-0.1: «««
-0.1 ≤ K-S Score<0: ««
K-S Score ≥ 0 or *p-value *≥ 0.05: « Click here for file
